# Ultrasound Elastography in the Diagnosis and Management of Uterine Pathologies: A Systematic Review

**DOI:** 10.3390/jcm15124468

**Published:** 2026-06-09

**Authors:** Sofia Bigardi, Orazio De Tommasi, Marta Tripepi, Emma Facchetti, Matteo Marchetti, Marco Noventa, Carlo Saccardi, Roberto Tozzi, Giulia Spagnol

**Affiliations:** Unit of Gynecology and Obstetrics, Department of Women and Children’s Health, University of Padua, 35122 Padua, Italy; orazio.detommasi@studenti.unipd.it (O.D.T.); marta.tripepi@studenti.unipd.it (M.T.); emma.facchetti@studenti.unipd.it (E.F.); matteo.marchetti@unipd.it (M.M.); carlo.saccardi@unipd.it (C.S.); roberto.tozzi@unipd.it (R.T.); giulia.spagnol@unipd.it (G.S.)

**Keywords:** ultrasound elastography, shear wave elastography, strain elastography, uterine pathologies, adenomyosis, uterine fibroids, cervical cancer, endometrial cancer

## Abstract

**Background/Objectives:** Ultrasound elastography (UE) is a non-invasive imaging technique that evaluates tissue stiffness and may complement conventional ultrasound in the assessment of uterine diseases. This systematic review aimed to summarize the current evidence on the role of strain elastography (SE) and shear wave elastography (SWE) in the diagnosis and management of benign and malignant uterine pathologies. **Methods:** A systematic literature search of MEDLINE (PubMed) and Embase was performed to identify studies published between January 2018 and February 2026. Original studies evaluating UE in adenomyosis, uterine fibroids, cervical lesions, and endometrial pathologies were included. Data were qualitatively synthesized according to pathology type and elastographic technique. **Results:** Twenty studies met the inclusion criteria. In benign myometrial disorders, adenomyosis and uterine fibroids generally showed higher stiffness than normal myometrium, although differentiation between these entities was not always consistent across studies. In cervical disease, malignant and high-grade lesions typically demonstrated increased stiffness compared with benign or low-grade lesions. In endometrial pathology, endometrial carcinoma was generally associated with higher stiffness values than benign lesions and elastography also showed potential in assessing myometrial invasion. Across studies, UE demonstrated promising diagnostic performance, but substantial heterogeneity was observed in acquisition methods, parameters, and reported thresholds. **Conclusions:** UE appears to be a promising adjunct to conventional ultrasound for the evaluation of uterine pathologies. However, further standardized, large-scale studies are needed to define reproducible protocols and clinically applicable diagnostic thresholds.

## 1. Introduction

Ultrasound (US) is currently the first-line imaging method for evaluating a wide range of pathological and physiological conditions affecting the female reproductive organs [1]. By utilizing high-frequency sound waves to create detailed images of internal structures, ultrasound offers a non-invasive, safe, and relatively cost-effective method for diagnosing and monitoring gynecological pathologies. Since its introduction into clinical practice in the 1970s, ultrasound technology has evolved significantly with the development of additional imaging modalities such as Doppler imaging, enhancing diagnostic capabilities [2]. Ultrasound elastography (UE) is an advanced imaging technique developed in the 1990s to non-invasively assess tissue biomechanical properties, providing images that reflect tissue rigidity or stiffness, mimicking the palpation performed by clinicians [2,3]. Elastography quantitatively evaluates tissue stiffness using Young’s modulus (E), a physical parameter that reflects tissue elasticity and varies significantly among different biological tissues, thereby enabling improved contrast and precise tissue characterization [4].

**Figure 1:**
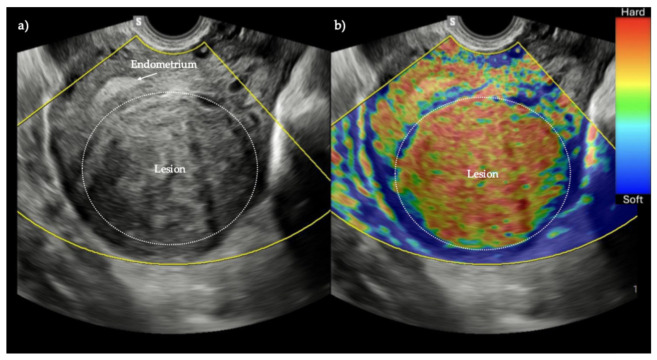
Application of SWE to a uterine lesion. (**a**) Grayscale ultrasound image of a uterine solid lesion (white dotted circle), with regular borders, homogeneous echogenicity and the presence of shadowing (leiomyoma on final histology). (**b**) SWE is applied to the same lesion. On the color-coded maps, hard tissues with high strain appear in red, while soft tissues with low strain appear in blue, with intermediate stiffness depicted in green. In this case, the uterine lesion appears predominantly stiff and rigid. On the right side of the image, a color-coded scale illustrates the appearance of hard and soft tissue on elastography. Please note that many systems allow customization of the color-map.

Elastography techniques are broadly categorized into two main groups: strain imaging and shear wave imaging [2,5], both of which require mechanical excitation to assess tissue properties. Strain elastography (SE) measures tissue deformation in response to externally applied pressure, typically using an ultrasound probe. The resulting elastogram consists of a conventional grayscale ultrasound image and a color-coded stiffness map. The strain ratio (SR) is a semiquantitative value calculated as the ratio between strain in a target region of interest (ROI) and that in a reference region. An SR greater than 1 suggests that the reference region is more deformable than the target region; however, the selection of an appropriate reference remains challenging [2]. Shear wave elastography (SWE) quantifies tissue stiffness by measuring the propagation speed of shear waves generated by acoustic radiation force impulses, thereby eliminating the need for external mechanical compression. SWE produces quantitative data expressed in kilopascals (kPa) or meters per second (m/s) and generates color-coded stiffness maps. Unlike SE, SWE enables independent calculation of stiffness in multiple regions, offering improved reproducibility and reduced operator dependency [5]. Acoustic radiation force impulse (ARFI) elastography, a form of SWE, allows point measurements of stiffness within a single ROI but does not permit simultaneous assessment of multiple regions, thus precluding stiffness ratio calculations (Figure 1). Both SE and SWE have demonstrated clinical utility in gynecological pathologies, with SWE offering superior quantitative capabilities and SE providing a more accessible option for qualitative assessments [2,4,5]. In this context, this simple and intuitive relationship between palpation and elastography calls for many applications of this “palpation imaging” such as breast tumor characterization and hepatic fibrosis staging where it has successfully been validated [5,6,7]. These techniques are now increasingly applied to gynecological conditions, as many pathological processes alter uterine tissue elasticity. Among elastographic modalities used in obstetrics and gynecology, SWE is currently the most widely adopted technique, as it can theoretically assess tissue stiffness at depths of up to 8 cm without requiring operator-applied compression [8]. The resulting imaging reflects the microstructural composition and organization of uterine pathology, increasing diagnostic accuracy similarly to how palpation aids gynecologic examination [9]. In benign conditions, such as uterine fibroids and adenomyosis, elastography has shown promising potential in characterizing tissue properties and guiding clinical management. In malignant conditions, including cervical and endometrial cancers, elastography has been explored as a tool to differentiate benign from malignant lesions and predict tumor aggressiveness. However, despite its potential, the integration of elastography into routine gynecological practice remains limited, due to variability in technique, lack of standardized parameters, and heterogeneity in the literature [10,11,12,13].

This systematic review aims to provide in-depth analysis of the current applications of UE in benign and malignant uterine pathologies. By summarizing available evidence, identifying key diagnostic parameters, and comparing the performance of different elastography techniques, we aim to highlight the clinical utility of elastography and guide future research in this field.

## 2. Materials and Methods

**Figure 2:**
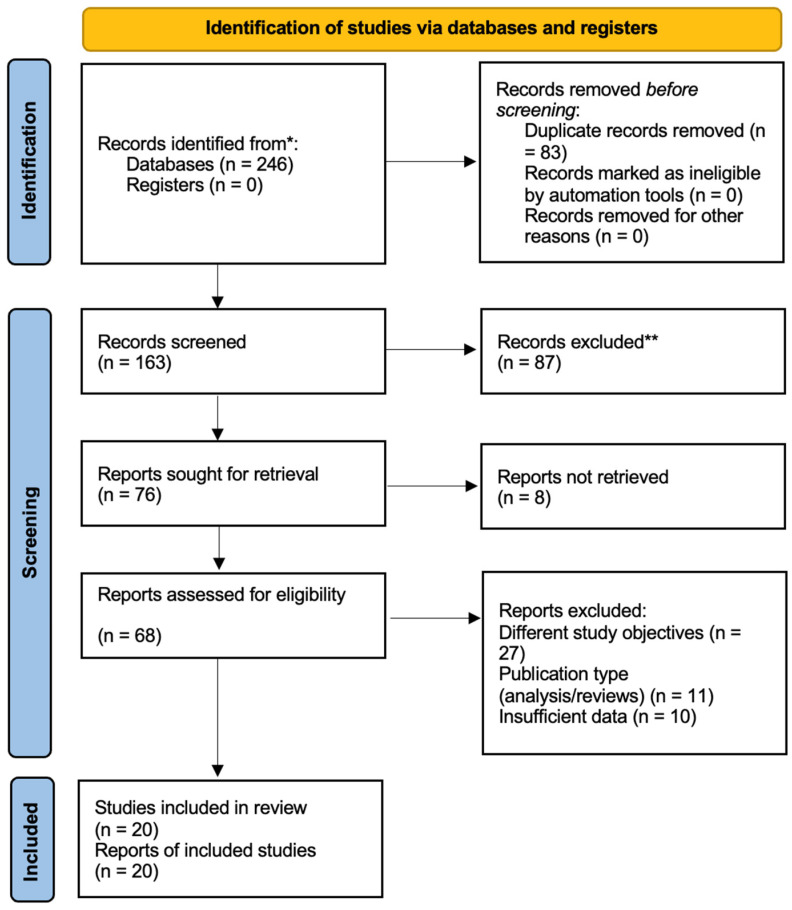
The flowchart of the screening process. * Consider, if feasible to do so, reporting the number of records identified from each database or register searched (rather than the total number across all databases/registers). ** If automation tools were used, indicate how many records were excluded by a human and how many were excluded by automation tools.

A systematic review was conducted and reported in accordance with the Preferred Reporting Items for Systematic Reviews and Meta-Analyses (PRISMA) statement. The PRISMA checklist is provided in the Supplementary Materials, and the study selection process is summarized in the PRISMA flow diagram (Figure 2). The review protocol was not prospectively registered. A systematic search of the literature was performed in MEDLINE (via PubMed) and Embase to identify relevant studies published from January 2018 to February 2026. This time frame was selected to focus on the most recent evidence and technological developments in ultrasound elastography applied to uterine pathology. The search strategy combined Medical Subject Headings (MeSH) in MEDLINE, Emtree terms in Embase, and relevant free-text terms. The following terms and their combinations were used: (“ultrasound elastography” OR “shear wave elastography” OR “strain elastography”) AND (“benign gynecologic pathology” OR “gynecologic malignancy” OR “adenomyosis” OR “uterine fibroids” OR “endometrial lesions” OR “cervical lesions”). Only studies published in English were considered. Eligible studies were original research articles evaluating the application of elastography in benign and malignant uterine pathologies. Case reports, conference abstracts, and reviews without original data were excluded. Two independent reviewers screened the titles and abstracts of the retrieved records to assess eligibility. Full-text articles of potentially relevant studies were then reviewed for final inclusion. Any disagreement between reviewers was resolved through discussion with a third reviewer. The following data were extracted from the included studies: study design, population characteristics, elastography technique used (SE or SWE), pathology evaluated, elasticity measurements (e.g., stiffness expressed in kPa or m/s), diagnostic performance metrics (e.g., sensitivity, specificity, and AUC), and main findings. In addition, the reference lists of the included studies were manually screened to identify any further relevant articles. Given the heterogeneity of study designs, elastographic parameters, and reported outcomes, a qualitative synthesis of the available evidence was performed.

## 3. Results

A total of 246 records were initially identified through database searching. After the removal of 83 duplicate records, 163 articles remained and were screened based on title and abstract. Following the initial screening process, 87 studies were excluded because they were not directly related to the clinical application of ultrasound elastography in uterine pathologies or did not meet the predefined inclusion criteria. Seventy-six full-text articles were sought for retrieval, and 8 could not be retrieved. Therefore, 68 full-text articles were assessed for eligibility. Of these, 48 were excluded because of different study objectives (n = 27), publication type (n = 11), or insufficient data (n = 10). Ultimately, 20 original studies were included in the final qualitative synthesis. The included studies comprised prospective, retrospective, and pilot studies evaluating the clinical application of ultrasound elastography in benign and malignant uterine pathologies. For clarity, the included studies were categorized according to the anatomical site evaluated. Six studies focused on benign myometrial disorders, including adenomyosis and uterine fibroids; six studies investigated cervical lesions, including cervical intraepithelial neoplasia (CIN) and cervical cancer (CC); and eight studies evaluated endometrial pathologies, including endometrial hyperplasia and endometrial carcinoma. Across the included studies, SE and SWE were used to assess tissue stiffness. While SE provides semiquantitative measurements based on strain ratio, SWE allows quantitative assessment of tissue elasticity expressed in kilopascals (kPa) or meters per second (m/s). Overall, most studies reported altered biomechanical properties in pathological uterine tissues compared with normal uterine tissue, supporting the potential role of elastography as a complementary diagnostic imaging technique.

### 3.1. Benign Myometrial Disorders: Adenomyosis and Uterine Fibroids

Ultrasound elastography has emerged as a promising imaging technique for the evaluation of benign myometrial disorders, particularly adenomyosis and uterine fibroids. These conditions are characterized by alterations in the microstructural composition of the myometrium, including increased fibrosis, smooth muscle proliferation, and extracellular matrix deposition, which may lead to measurable differences in tissue stiffness.

Several clinical studies have demonstrated that elastography can effectively detect stiffness differences between normal myometrium and pathological uterine tissues (Table 1). Liu et al. [14] reported that adenomyotic lesions exhibited significantly higher stiffness values compared with normal myometrium and were approximately 2.6 times stiffer than uterine fibroids, and both were stiffer than normal myometrium. The study also revealed that lesional stiffness correlated positively with the extent of fibrosis and the severity of symptoms, suggesting that TVESG could be used not only for diagnosis but also for assessing disease severity and guiding treatment decisions. Recently a similar result was support by a pilot study which identified significantly higher mean SR and maximum SR values among both adenomyosis (AM) and uterine fibroid (UF) lesions as opposed to controls (*p* < 0.01), with the highest tissue stiffness being encountered among AM lesions, which allows for the differentiation of UF (*p* < 0.01) and concomitant identification of both lesions with higher cut-off values obtained for AM [15]. Further supporting the utility of elastography, Zhang M et al. [16] observed increased shear wave velocities (SWVs) in both AM and UF compared with normal myometrium, although the technique was not always able to clearly distinguish between the two entities. These partially discordant findings compared with previous studies may reflect methodological differences, particularly the use of different elastographic approaches, variations in ROI selection and lesion sampling, and heterogeneity in tissue composition, fibrosis, and patient characteristics. This suggests that while elastography adds value, it may need to be combined with other diagnostic tools or techniques for optimal differentiation. Pongpunprut et al. [17] expanded on this by comparing shear wave velocity across normal myometrium, fibroids, and adenomyosis. They identified reported significantly higher shear wave velocities in adenomyosis compared with normal myometrium, with a proposed cutoff value of 3.465 m/s for identifying adenomyosis.

Beyond diagnostic differentiation, elastography may also provide insights into disease severity and pathophysiology. Ren et al. [18] demonstrated a strong correlation between lesion stiffness and the severity of dysmenorrhea in patients with adenomyosis. Moreover, in women with adenomyosis, lesional stiffness has been found to be correlated with severity of dysmenorrhea and the amount of menstrual blood loss (MBL) and can be used to distinguish adenomyotic lesions from uterine fibroids [14].

More recently, pilot studies have explored the feasibility of measuring uterine tissue stiffness using advanced elastographic techniques. Ryles et al. [19] conducted a protocol pilot study investigating transvaginal shear wave elastography for assessing myometrial and leiomyoma stiffness, demonstrating the feasibility of quantitative stiffness measurements in uterine tissues and supporting the potential role of elastography in the characterization of UF. Overall, current evidence suggests that ultrasound elastography may represent a valuable adjunct to conventional ultrasound in the evaluation of benign myometrial disorders. By providing quantitative information about tissue stiffness, elastography may improve the differentiation between adenomyosis and uterine fibroids and contribute to a better understanding of the biomechanical characteristics of these conditions.

### 3.2. Malignant Uterine Pathologies

#### 3.2.1. Cervical Lesions

Ultrasound elastography has emerged as a promising imaging tool for the evaluation of cervical pathologies, including cervical intraepithelial neoplasia (CIN), premalignant lesions and cervical cancer (CC). By providing quantitative and qualitative information on tissue stiffness, elastography offers valuable diagnostic and prognostic information.

Several clinical studies have demonstrated that malignant cervical lesions generally exhibit increased stiffness compared with benign or normal cervical tissue (Table 2). Liu et al. [20] reported significantly higher mean and maximum shear wave velocities in malignant cervical lesions compared with benign and normal tissues, achieving a high diagnostic performance for differentiating malignant from benign cervical disease. Elastography has also shown potential in the evaluation of premalignant cervical lesions. Sainz et al. [21] demonstrated significant differences in shear wave velocity between high-grade squamous intraepithelial lesions (HSIL) and low-grade lesions (LSIL), suggesting that elastography may contribute to the stratification of cervical epithelial abnormalities. In addition, strain elastography has been investigated as a method to differentiate CIN and invasive cancer from normal cervical tissue. In this context, Dudea-Simon et al. [22] reported promising diagnostic performance of strain ratio measurements in distinguishing cervical pathology from normal tissue, using an experimental synthetic device as a reference. More recently, Yücel et al. [23] investigated the role of cervical elastography in differentiating preinvasive from invasive cervical lesions and reported that elastographic parameters may significantly aid in distinguishing early-stage cervical neoplasia from invasive disease, supporting its potential use as a complementary diagnostic tool in cervical cancer assessment.

In addition to its diagnostic value, ultrasound elastography has also been explored as a potential tool for prognosis assessment and treatment monitoring in cervical cancer. Changes in tissue stiffness during therapy may reflect modifications in tumor structure and response to treatment. Zhang Y et al. [24] demonstrated that the sensitivity, specificity and diagnostic accordance rate of elastic ultrasound SR value in the efficacy evaluation of radiotherapy in CC patients were higher than those of conventional ultrasound. Similarly, Xu et al. [25] demonstrated that SE parameters, such as strain ratio and tumor diameter changes during concurrent chemoradiotherapy (CCRT), could predict long-term outcomes in patients with advanced CC. This indicates elastography’s role as a non-invasive tool to monitor treatment response.

#### 3.2.2. Endometrial Lesions

Ultrasound elastography has increasingly been investigated as a non-invasive imaging technique for the evaluation of endometrial pathologies, including endometrial cancer, endometrial hyperplasia, and benign lesions such as polyps.

Many studies have demonstrated that malignant endometrial lesions generally exhibit higher stiffness compared with benign conditions or normal endometrium (Table 3). Che et al. [26] as well as Ong CL et al. [27] reported that strain values obtained through transvaginal elastography showed good diagnostic performance in distinguishing endometrial cancer from benign endometrial masses. Similarly, Du et al. and Ma H et al. [28,29] found that shear wave elastography parameters, including mean and maximum elasticity values, were significantly higher in endometrial carcinoma compared with proliferative or secretory endometrium, endometrial polyps, and endometrial hyperplasia. Vora et al. [30] conducted a prospective pilot study assessing SWE in endometrial and subendometrial pathologies. They reported significant differences in mean, minimum, and maximum elasticity values among different pathologies (*p* < 0.001). Notably, endometrial polyps exhibited lower mean elasticity compared to other lesions, while submucosal leiomyomas and focal adenomyomas displayed significantly higher elasticity values (*p* < 0.01). However, no significant difference was found in the elasticity of carcinoma and hyperplasia (*p* = 0.19), indicating a potential overlap in these conditions. On the other hand, recent investigations have also explored the role of elastography in identifying early or precancerous endometrial alterations. Ma H and Recently Guler et al. [31] showed that shear wave elastography may contribute to predicting endometrial cancer in women presenting with abnormal uterine bleeding, while Zorlu et al. [32] demonstrated that elastographic parameters may assist in the non-invasive diagnosis of endometrial hyperplasia, potentially improving patient triage and reducing unnecessary invasive procedures. In addition to diagnosis, elastography may also provide valuable information regarding tumor characteristics and depth of myometrial invasion. Zhao HX et al. [33] demonstrated that real-time shear wave elastography could differentiate superficial from deep myometrial invasion in endometrial cancer with high diagnostic accuracy. These findings highlight the potential role of elastography not only in lesion detection but also in staging and treatment planning.

## 4. Discussion

This comprehensive review highlights the increasing role of ultrasound elastography, particularly SWE and SE, in the diagnosis and evaluation of uterine pathologies. Transvaginal ultrasound remains the first-line imaging technique for evaluating uterine pathology, as it allows accurate assessment of uterine morphology, including the size, number, and location of fibroids. In adenomyosis, TVUS may reveal characteristic findings such as myometrial cysts, heterogeneous myometrial texture, and a globular enlarged uterus. However, conventional imaging methods are limited in their ability to evaluate tissue mechanical properties, which may reflect the degree of fibrosis and structural remodeling within uterine lesions. From a pathophysiological perspective, both adenomyosis and uterine fibroids are characterized by substantial extracellular matrix deposition and progressive fibrosis. Adenomyotic lesions undergo processes such as epithelial–mesenchymal transition, fibroblast-to-myofibroblast transdifferentiation, and smooth muscle metaplasia, leading to progressive tissue remodeling and increased stiffness. Similarly, fibroids exhibit abundant extracellular matrix accumulation and smooth muscle proliferation, which significantly alters the mechanical behavior of the affected tissue. Because the extent of fibrosis directly influences tissue rigidity, elastography can provide indirect information about the structural composition and progression of uterine lesions. By providing quantitative or semiquantitative information on tissue stiffness, elastography represents a valuable complement to conventional ultrasound, allowing the assessment of biomechanical properties that are not detectable with standard imaging techniques.

The studies included in this review consistently demonstrate that pathological uterine tissues exhibit increased stiffness compared with normal myometrium. In benign uterine conditions, elastography has shown promising potential for distinguishing adenomyosis and uterine fibroids from surrounding normal tissue. Recent literature reviews and meta-analyses further support the diagnostic value of elastography in benign uterine diseases. Brunelli et al. [10] conducted a systematic review and meta-analysis evaluating the role of ultrasound elastography in the diagnosis of endometriosis and adenomyosis and concluded that elastographic techniques show promising diagnostic performance in identifying adenomyotic lesions. Similarly, Guo et al. [9] highlighted the potential role of elastography in developing imaging-based classifications of adenomyosis, suggesting that stiffness measurements could contribute to a more objective characterization of disease phenotypes.

In addition to its diagnostic role, elastography may also provide insights into disease severity and symptomatology. Increased stiffness in adenomyotic lesions has been associated with greater degrees of fibrosis and with clinical manifestations such as dysmenorrhea and abnormal uterine bleeding. These findings suggest that elastography may have potential applications not only in diagnosis but also in evaluating disease severity and monitoring treatment response. Interestingly, lesion stiffness may also be associated with hormonal responsiveness. Lesions characterized by lower stiffness values often show higher vascularity and progesterone receptor expression, which may predict a better response to hormonal therapies such as dienogest. Conversely, highly fibrotic lesions with increased stiffness may demonstrate reduced hormonal responsiveness [34,35]. These observations suggest that elastography could potentially contribute to individualized therapeutic strategies by identifying patients who are more likely to benefit from medical treatment.

Despite these promising results, the differentiation between adenomyosis and uterine fibroids remains challenging in some cases. The partially discordant findings regarding the relative stiffness of adenomyosis and fibroids likely reflect substantial heterogeneity across studies, including differences in elastography technique, ROI placement, acquisition protocols, lesion composition, degree of fibrosis, and patient population, all of which may influence stiffness measurements and limit direct comparability.

The application of elastography in malignant uterine pathologies has also shown encouraging results. In cervical disease, elastography has demonstrated the ability to differentiate malignant lesions from benign cervical tissue and premalignant abnormalities such as cervical intraepithelial neoplasia. Several studies have reported higher stiffness values in cervical cancer compared with benign cervical lesions, supporting the potential role of elastography in improving diagnostic accuracy. A meta-analysis by Zhu et al. [36] reported that transvaginal sonoelastography demonstrates good diagnostic accuracy in distinguishing malignant from benign cervical lesions, supporting its potential role as a complementary imaging modality in gynecologic oncology. Likewise, broader reviews have summarized the technological development and clinical applications of elastography in cervical imaging. Shao et al. [37] described the expanding role of elastography in the assessment of cervical infiltration depth, emphasizing its potential utility for diagnosis, staging, and monitoring treatment response. Similarly, a systematic review by Dudea-Simon et al. [11] provided an overview of elastographic findings in the normal cervix and in pathological conditions such as CIN and cervical cancer, further reinforcing the growing clinical interest in this technique. More recently, a comprehensive systematic review and meta-analysis by Mahdavi Sabet et al. [38] evaluated the diagnostic performance of ultrasound elastography in cervical neoplasms and confirmed that elastographic techniques demonstrate promising sensitivity and specificity in differentiating malignant from benign cervical lesions. These findings support the potential integration of elastography into multiparametric imaging approaches for cervical cancer evaluation.

Moreover, elastography may also provide prognostic information in cervical cancer. Changes in elastographic parameters during treatment have been associated with tumor response to therapy, suggesting that elastography may serve as a non-invasive imaging biomarker for monitoring treatment outcomes in patients undergoing chemoradiotherapy. However, Zhang HP [39] emphasized the importance of strict quality control in 2D SWE to ensure reproducibility. Factors such as age and cervical position significantly influenced cervical stiffness readings, whereas menstrual cycle and HPV status did not. This underscores the need to standardize elastography protocols to minimize variability and enhance reliability. These findings collectively suggest that elastography can enhance the diagnostic, staging, and prognostic evaluation of cervical pathologies. However, further large-scale studies are needed to refine cutoff values, establish standardized protocols, and integrate elastography into routine clinical practice.

Similarly, elastography has shown potential in the evaluation of endometrial pathologies. Several studies have reported higher stiffness values in endometrial carcinoma compared with benign endometrial lesions such as polyps or hyperplasia. In addition to diagnostic applications, elastography may also provide useful information regarding tumor characteristics, including the depth of myometrial invasion, which represents an important prognostic factor in endometrial cancer. Evidence from meta-analyses further supports these findings. In 2023 Bian et al. [12] performed a meta-analysis evaluating the diagnostic accuracy of shear wave elastography for endometrial cancer and reported promising pooled sensitivity and specificity, confirming the potential of elastography as a complementary diagnostic tool in the assessment of endometrial malignancies.

Furthermore, studies have also focused on the effects of tamoxifen on endometrial tissue. Tamoxifen is a commonly used selective estrogen receptor modulator applied in the treatment for breast cancer. However, in the endometrium, Tamoxifen stimulates tissue growth, cellular transformation, the migration of the cells, and metastatic potential in endometrial cancer. Studies by Jabłoński et al. and Jo et al. [40,41] investigate if applying elastography in examining the endometrium was beneficial for uterine cancer screening protocols. In this contest Elastography may provide additional insights into endometrial changes induced by tamoxifen, enhancing cancer screening protocols for patients undergoing treatment. In particular Jabłoński et al. [40] investigated elastography in the context of endometrial assessments for women undergoing tamoxifen treatment. This study suggests a novel approach to diagnosing endometrial changes associated with tamoxifen, providing insights into its effects on endometrial elasticity.

Despite these promising results, variability in acquisition techniques, elastographic parameters, and cutoff values remains a major limitation across studies. Standardization of imaging protocols and larger prospective studies are therefore required to fully establish the role of elastography in the diagnostic work-up of endometrial diseases.

Despite the growing body of evidence supporting the clinical utility of elastography, several limitations remain. Considerable heterogeneity exists among the available studies with respect to elastography techniques, ultrasound equipment, measurement protocols, and reported stiffness parameters. The absence of standardized acquisition protocols and universally accepted cutoff values limits the comparability of results across studies and currently represents one of the major obstacles to the widespread clinical adoption of elastography. Furthermore, many studies included relatively small patient populations, which reduces the statistical power of the reported findings. Technical limitations should also be considered, as ultrasound signals may attenuate in deep tissues, potentially reducing the accuracy of elastographic measurements for deeply located or very small lesions. Operator dependency represents another important limitation, particularly for strain elastography, where the degree of compression applied by the transducer can influence the measured stiffness values. In contrast, shear wave elastography provides quantitative stiffness measurements and is generally considered less operator-dependent. Nevertheless, both techniques require careful standardization to ensure reproducibility and reliability across different clinical settings.

### Clinical Implications and Future Perspectives

The integration of elastography into gynecological imaging has the potential to expand the diagnostic capabilities of conventional ultrasound by introducing a functional assessment of tissue biomechanics. By quantifying differences in tissue stiffness, elastography may improve the characterization of uterine lesions and support more accurate differentiation between benign and malignant conditions. Future developments may include the establishment of standardized elastography protocols, including the choice of elastographic parameters, ROI selection, and the definition of reliable stiffness cutoff values for different uterine pathologies. In addition, integrating elastographic measurements with clinical variables and conventional imaging findings may allow the development of predictive models or diagnostic nomograms to assist clinical decision-making. Large multicenter prospective studies will be essential to validate the diagnostic and prognostic role of elastography and to determine its optimal integration into routine gynecological practice. With further technological advancements and methodological standardization, ultrasound elastography may become an important component of multiparametric imaging strategies for the evaluation and management of uterine diseases.

## 5. Conclusions

Ultrasound Elastography represents a valuable tool in the evaluation of uterine pathologies, offering high sensitivity and specificity for distinguishing benign and malignant lesions. For benign conditions like adenomyosis and fibroids, elastography not only enhances diagnostic accuracy but also provides insights into disease severity and symptomatology. In malignant lesions such as cervical and endometrial cancers, elastography shows potential for both diagnosis and staging, with stiffness values correlating with disease progression. However, limitations related to operator dependency, challenges in assessing deep or small lesions, and the lack of standardized protocols must be addressed to optimize its clinical utility. Additionally, future research should focus on larger, multicenter studies to validate the findings and expand the use of elastography in monitoring treatment response and disease progression. The findings consistently underscore the value of elastography in enhancing diagnostic accuracy, providing insights into disease severity, and aiding in treatment planning. Elastography represents a promising, non-invasive adjunct to traditional diagnostic modalities, with the potential to revolutionize the management of uterine pathologies by enabling personalized, targeted treatment approaches.

## Figures and Tables

**Table 1:** Elastography Parameters for Benign Uterine Pathologies.

Year	Author	Study Design	N	Elastography Parameter	Lesion Type	Stiffness Values	Control Values	Cut-Off	Sensitivity (%)	Specificity (%)	AUC
2018	Liu X et al. [14]	Prospective	54	SWE (kPa)	UF AM	2.68 ± 0.66 3.90 ± 1.00	1.54 ± 0.82	NP	NP	NP	NP
2019	Zhang M et al. [16]	Prospective	34	SWV (m/s)	UF AM	5.6 ± 2.5 4.9 ± 2.5	4.8 ± 1.9	NP	NP	NP	NP
2022	Pongpunprut S et al. [17]	Prospective	75	SWV (m/s)	AM UF	4.63 ± 1.45 4.53 ± 1.07	3.44 ± 0.95	3.46	80	80	0.80
2022	Săsăran V et al. [15]	Prospective	63	SE (SR)	AM UF	11.52 ± 1.80 5.63 ± 1.14	1.49 ± 0.18	5.42 2.85	100	100	1.00
2023	Ren Q et al. [18]	Prospective	96	SE (SR)	AM	2.04 (1.71–2.25)	1.02 (0.96–1.09)	1.36	94.7	100	0.98
2026	Ryles HT et al. [19]	Pilot study	NP	NP	UF	NP	NP	NP	NP	NP	NP

SE: Strain Elastography; SR: Strain Ratio; SWE: Shear wave elastography; SWV: shear wave velocities AM: Adenomyosis; UF: Uterine Fibroids; NP: Not provided.

**Table 2:** Elastography Parameters for Malignant Cervical Lesions.

Year	Author	Study Design	N	Elastography Parameter	Lesion Type	Stiffness Values	Control Values	Cut-Off	Sensitivity (%)	Specificity (%)	AUC
2019	Liu C et al. [20]	Retrospective	246	SWV (m/s)	Malignant Benign	4.91 ± 1.12 3.53 ± 0.52	2.86 ± 0.23	3.25 m/s	90.9	87.8	NP
2019	Zhang Y et al. [24]	Prospective	160	SE (SR)	CC	NP	NP	NP	94.67	92.94	NP
2020	Dudea-Simon M et al. [22]	Prospective	79	SE (SR)	CIN CC	1.42 2.07	0.89	1.03 1.42	75 100	74 94.9	NP
2020	Xu Y et al. [25]	Prospective	68	SE (SR)	CC	NP	NP	NP	NP	NP	NP
2021	Sainz JA et al. [21]	Prospective	96	SWV (m/s)	HSIL LSIL	4.1	3.0	3.25	62.5	75.5	NP
2024	Yücel, Ecem et al. [23]	Prospective study	95	SE	LSIL HSIL CC	NP	NP	NP	94.7	96.1	NP

SE: Strain Elastography; SR: Strain Ratio; SWVs: shear wave velocities; CC: Cervical cancer; CIN: Cervical Intraepithelial Neoplasia; HSIL: High-Grade Squamous Intraepithelial Lesion; LSIL: Low-Grade Squamous Intraepithelial Lesion; NP: Not provided.

**Table 3:** Elastography Parameters for Malignant Endometrial Lesions.

Year	Author	Study Design	N	Elastography Parameter	Lesion Type	Stiffness Values	Control Values	Cut-Off	Sensitivity (%)	Specificity (%)	AUC
2019	Che D et al. [26]	Prospective	217	SE (SR)	EC	4.1 ± 1.3	2.2 ± 0.8	3.02	81.7	85	0.91
2021	Zhao HX et al. [33]	Prospective	108	SWE (kPa)	EC	39.4	23.8	39.03	92.9	97.3	0.95
2021	Du YY et al. [28]	Prospective	140	SWE (kPa)	Polyps EH EC	15.68 21.20 49.36	26.24	28.50	83.3	98	0.87
2021	Ma H et al. [29]	Prospective	120	SWE (kPa)	EC AEH	59.49 38.46	29.80– 17.96	NP	NP	NP	NP
2022	Ong CL et al. [27]	Prospective	79	SE (SR)	EC	Higher	Lower	NP	NP	NP	NP
2022	Vora Z et al. [30]	Prospective	73	SWE (kPa)	EH EC SL EP FA	23.14 26.60 56.81 12.25 49.01	NP	NP	NP	NP	NP
2024	Guler AH et al. [31]	Prospective	86	SWE (kPa)	EC	17.14	10.39–11.49	NP	NP	NP	NP
2025	Zorlu et al. [32]	Prospective	235	SWE (kPa)	AEH	37.71	NP	NP	85.9	48.0	NP

SE: Strain Elastography; SR: Strain Ratio; SWE: Shear wave elastography; EH: Endometrial Hyperplasia; SL: Submucosal leiomyoma, EP: Endometrial polyp; EC: Endometrial cancer; FA: Focal adenomyoma; NP: Not provided.

## Data Availability

No new datasets were generated in this study. The data supporting the findings of this systematic review are derived from previously published articles, which are cited in the reference list.

## Supplementary Materials

The following supporting information can be downloaded at: https://www.mdpi.com/article/10.3390/jcm15124468/s1, File S1: PRISMA_2020_abstract_checklist; File S2: PRISMA_2020_flow_diagram.
